# Supplementary material for: Estrogens Play a Critical Role in Stress-Related Gastrointestinal Dysfunction in a Spontaneous Model of Disorders of Gut–Brain Interaction
Source: Cells. 2022 Apr 4;11(7):1214. doi: 10.3390/cells11071214 (PMC8997409; doi:10.3390/cells11071214)
Supplement: Supplementary file 1 [file cells-11-01214-s001.zip › cells-1628051-supplementary.pdf]

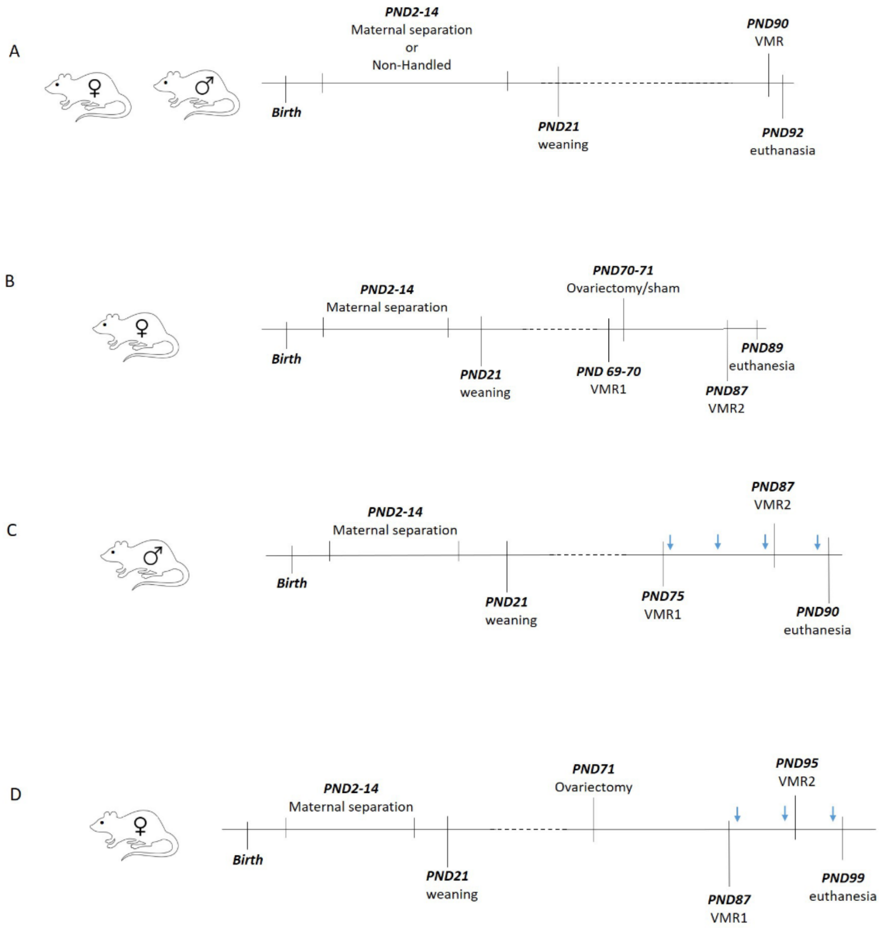


**Supplementary figure S1: Overview of the protocols**. Protocol 1 (A) applied in both males and females BBDPN rats; protocol 2 applied on the MS females (B & D) and for the MS males (C). Blue arrows: subcutaneous injections of β-estradiol 3-benzoate or vehicle. VMR: visceromotor response; BBDP-N: BioBreeding diabetes prone normoglycemic rats; PND: post-natal day; MS: maternal separation

**Supplementary table S1: qPCR primers**

| gene | **reverse** | **forward** |
|---|---|---|
| **F2rl1** | GCGTGTCCAATCTGCCAATC | TGCTGGGAGGTATCACCCTT |
| **mcpt2** | CATGAGCCACACCAGCACAC | GATGAAAAGGCCTGTGTGGACT |
| **ccl2** | CTAGTTCTCTGTCATACT | ATGCAGGTCTCTGTCACG |
| **ccl11** | AGATGCACGCTGAAAGCCATAGTC | GGTGCCGATATTCTCCCATAGCAT |
| **il-4** | CAGACGTCCTTACGGCAACA | AGCACGGAGGTACATCACGT |
| **gper1** | TGGTATGACTGCCTTGAGCG | CATGCCTACCCCTTGACAGG |
| **gapdh** | TCAATGAAGGGGTCGTTGATGG | TCTCTGCTCCTCCCTGTTCTAGAG |

*F2rl1: gene coding for par2 (protease-activated receptor 2); mcpt2: mast cell protease 2; ccl2: gene coding for mcp1 (monocyte chemoattractant protein1); ccl11: gene coding for eotaxin-1; il4: interleukin 4; gper: G-protein coupled estrogen receptor; gapdh: D-glyceraldehyde-3-phosphate dehydrogenase.*

**Supplementary table S2: Immune cells activation following ovariectomy in female and β–estradiol in males.**

| | **female** | | | **male** | | |
|---|---|---|---|---|---|---|
| | Sham | Ovx | *p* | vehicle | E2 | *p* |
| Jejunum EPO | 15(6.1-61.6) *n=6* | 38.5(3-76.6) *n=8* | 0.3 | 53.2(25.1-57.6) *n=4* | 37.6(0.8-56.4) *n=6* | 0.4 |
| Colon EPO | 0.3(0.2-0.5) *n=6* | 0.4(0.3-0.6) *n=6* | 0.3 | 0.2(0.1-0.2) *n=4* | 0.2(0.1-0.3) *n=6* | 0.6 |
| Jejunum β-hexoaminidase | 4.2(3.8-7.5) *n=7* | 7.5(4.6-8.2) *n=8* | 0.28 | 15.9(7.2-19.7) *n=4* | 8.6(4.2-10.7) *n=3* | 0.2 |
| colon β-hexoaminidase | 11.2(7.6-16) *n=7* | 14.4(6.2-35) *n=7* | 0.39 | 17.3(9.9-24.8) *n=5* | 13.6(12-27.3) *n=4* | 0.2 |

*EPO: eosinophil peroxidase; ovx: ovariectomy; E2: β–estradiol. Mann-Whitney t-test.*
